# Longitudinal trajectories and determinants of human fungiform papillae density

**DOI:** 10.18632/aging.203741

**Published:** 2021-12-02

**Authors:** Ajoy C. Karikkineth, Eric Y. Tang, Pei-lun Kuo, Luigi Ferrucci, Josephine M. Egan, Chee W. Chia

**Affiliations:** 1 Clinical Core Laboratory and Biorepository, Intramural Research Program, National Institute on Aging, National Institutes of Health, Baltimore, MD 21224, USA; 2 Laboratory of Clinical Investigation, National Institute on Aging, National Institutes of Health, Baltimore, MD 21224, USA; 3 Translational Gerontology Branch, National Institute on Aging, National Institutes of Health, Baltimore, MD 21224, USA

**Keywords:** fungiform papillae, taste, aging, longitudinal, taste buds

## Abstract

Tongue fungiform papillae contain taste buds crucial for taste and hormone-producing taste receptor cells; therefore, they may be considered as endocrine organs and have important age-associated physiological implications. We examine the cross-sectional and longitudinal trajectories of fungiform papillae density in 1084 participants from the Baltimore Longitudinal Study of Aging using linear regression models and mixed effects models. At baseline, the mean age was 67.86 ± 14.20 years, with a mean follow-up time among those with repeat visits of 4.24 ± 1.70 years. Women (53%) were younger (66.85 ± 13.78 vs. 69.04 ± 14.61 years, *p* < 0.001) and had a higher fungiform papillae density than men (16.14 ± 9.54 vs. 13.77 ± 8.61 papillae/cm^2^, *p* < 0.001). Whites (67%) had a lower fungiform papillae density than non-Whites after adjusting for age and sex. Factors cross-sectionally associated with a lower fungiform papillae density included a higher waist-hip ratio (β = −8.525, p = 0.029), current smoking status (β = −5.133, p = 0.014), and alcohol use within the past 12 months (β = −1.571, p = 0.025). Longitudinally, fungiform papillae density decreased linearly with follow-up time (β = −0.646, p < 0.001). The rate of decline was not affected by sex, race, BMI, waist-hip ratio, smoking, or alcohol use. The longitudinal decline of fungiform papillae density over time needs to be explored further in order to identify other possible age-associated physiological determinants.

## INTRODUCTION

Taste is one of the five fundamental primary senses and plays a crucial role in our interactions with the environment. Increased interest in research related to both taste and smell has now been fueled by the present SARS-CoV-2 pandemic because as many as 80% of people infected report taste and smell loss and/or changes [1]. An unknown but increasing percentage of people with long-Covid syndrome also continue to report taste and smell changes.

**Figure 1:**
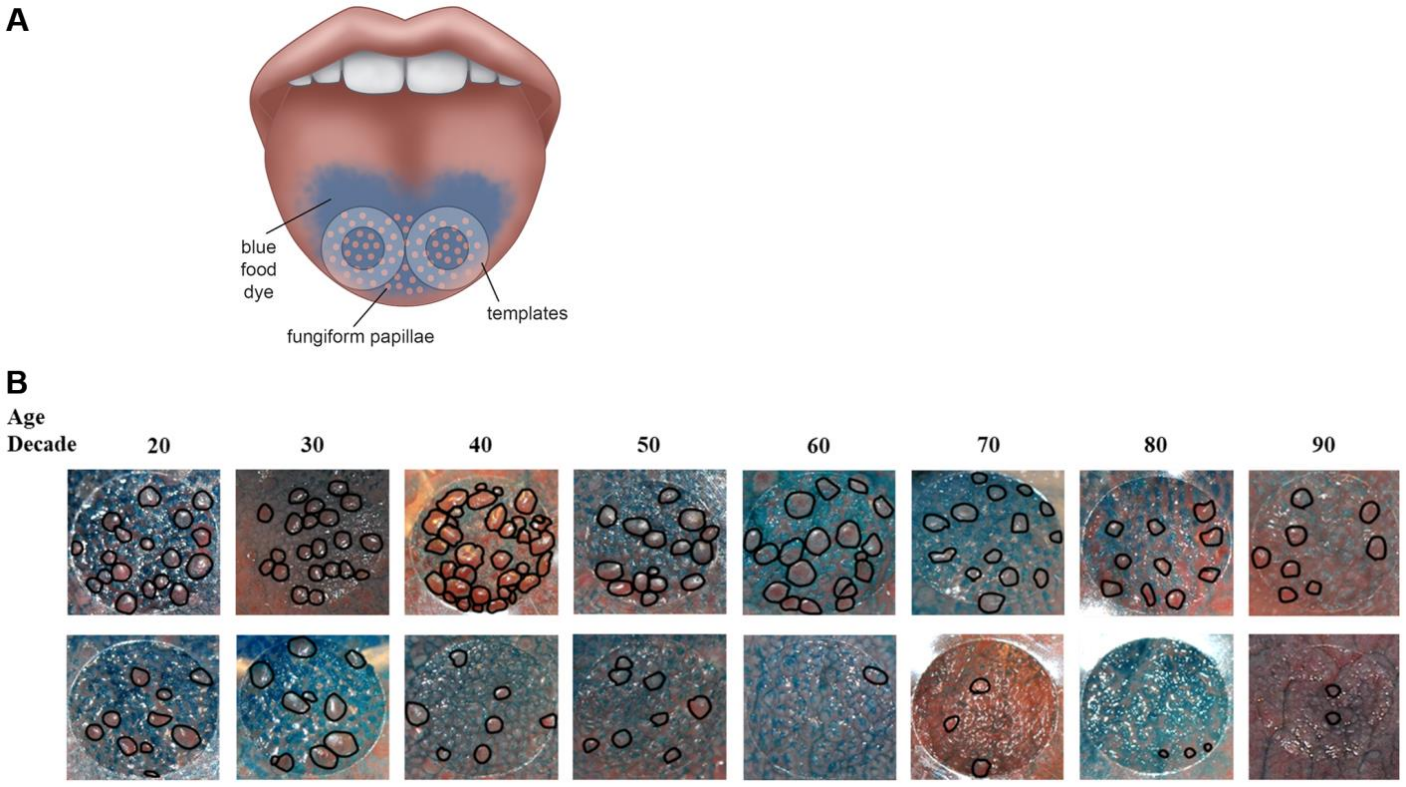
(**A**) Method to assess fungiform papillae density. Blue food coloring is used to provide optimal contrast between fungiform papillae (do not take up blue dye and appear pink) and other tongue structures (coated blue). Two clear plastic hole reinforcement templates (7 mm in diameter) are placed posterior to the apex of the tongue on each side of the median sulcus. Tongue images containing the two templates were taken using a digital camera. The fungiform papillae present within the two 7 mm holes were then counted and normalized to the area of the holes and expressed as fungiform papillae density (number of fungiform papillae/cm^2^). (**B**) Representative tongue images from 16 participants with age spans from the 20s to the 90s. As shown, fungiform papillae density varies widely among individuals and across lifespan. The top panel shows individuals with higher fungiform papillae density versus those individuals with lower fungiform papillae density in the lower panel.

Taste signaling begins in specialized chemosensory taste receptor cells within taste buds that are located within specialized structures called taste papillae on our tongues [2]. Fungiform papillae, one type of taste papillae, are densely packed on the anterior tongue (Figure 1A). There is a wide variation in the number of fungiform papillae across individuals, with reported estimates ranging from less than 10/cm^2^ to greater than 200/cm^2^ [3, 4]. The underlying reason for this wide variation is not clear. Studies in different populations have suggested that fungiform papillae density declines with aging. However, most of the published studies on the association of fungiform papillae density with age to date have been cross-sectional [4], and mostly in small study populations of less than a hundred individuals [5–7]. These studies are unable to distinguish between age related changes and cohort effects. One study which looked at longitudinal changes of fungiform papillae density examined a narrow age cohort, and was mainly designed to look at changes with obesity rather than longitudinal changes with age [8].

Understanding whether fungiform papillae density decreases with age is important because beyond their role in taste perception, which by itself can affect eating behavior, fungiform papillae play other important and still not elucidated physiological roles. For example, fungiform papillae provide a source of undifferentiated stem cells to replenish taste receptor cells as they are lost [9]. Therefore, physiologically it would seem very likely that a lower fungiform papillae density would lead to lower taste perception. A lower fungiform papillae density has been associated with lower taste intensity perception in both healthy individuals [3, 10] and in those with disease conditions [11]. The lack of association between fungiform papillae density and perceived taste perception in a recent study [4] is surprising, and may be due to methodological issues in assessing taste perception. Electrogustatory thresholds, which are more objective measures of taste perception than perceived taste intensity, are associated not only with the number of fungiform papillae but also with their structure and vascularization [12, 13]. A lower taste perception is an important issue because it impairs quality of life, and it has also been associated with poor dietary choices [14], obesity [15], increased rates of smoking [16], and alcohol use [17].

As our understanding of the physiological roles of fungiform papillae and taste receptors improve, it is becoming increasingly clear that the function of fungiform papillae, taste receptor cells, and taste receptors are intertwined with other physiological systems in the body. Taste receptors are expressed in extra-gustatory tissues where they serve various functions. The bitter taste receptor TAS2R38 has been shown to regulate differentiation and de-lipidation in adipocytes [18], and has been associated with Parkinson’s disease [19] and gastric cancer [20]. Additionally, hormone receptors normally associated with extra-gustatory tissues, and hormones such as CCK [21], glucagon [22], glucagon-like peptide-1 (GLP-1) [23, 24], ghrelin [25], VIP [26], and insulin [27], have been shown to be produced in taste receptor cells. Indeed, emerging research suggests that fungiform papillae may be considered as endocrine organs.

Few studies have examined factors that may affect the rate of longitudinal changes in fungiform papillae density with aging. For example, some studies have reported that male sex [4, 28], smoking [29], and alcohol use [4] are associated with a lower fungiform papillae density, but we do not know what role these factors play in the change in fungiform papillae density over time, or the direction of their associations. Racial differences have been reported in taste sensitivity [17, 30], but as far as we know, there are no studies reporting racial differences in fungiform papillae density either cross-sectionally or longitudinally. The associations between obesity and fungiform papillae density are also complicated. Both higher taste thresholds [16, 31] and increased taste sensitivity [32] have been shown to be associated with obesity. However, it has also been reported that mice and men who were obese had a loss of fungiform papillae, possibly mediated through inflammation [8]. Therefore, the exact direction of the associations between obesity, taste, and fungiform papillae density remains unclear. With fungiform papillae being considered as possible endocrine organs, the understanding of their age-associated changes can have important implications.

We studied fungiform papillae density in men and women from the Baltimore Longitudinal Study of Aging (BLSA) with the aims to: (1) delineate longitudinal patterns of change of fungiform papillae density; (2) explore sex and racial differences in the rate of change; and (3) identify other possible determinants of the rate of longitudinal changes in fungiform papillae density.

## RESULTS

Our study population included 1084 participants, 53 percent of whom were women (Table 1). The mean age at baseline was 67.86 ± 14.20 years, with a mean follow-up time among those with repeat visits of 4.24 ± 1.70 years (range 0.90 to 8.21 years) over 3.04 ± 1.18 visits (range 2 to 9 visits); 66.97% of the study population were Whites. Women were younger than men (66.85 ± 13.78 years vs. 69.04 ± 14.61 years, *p* < 0.001), and fungiform papillae density at baseline was higher among women than men (16.14 ± 9.54 papillae/cm^2^ vs. 13.77 ± 8.61 papillae/cm^2^, *p* < 0.001).

**Table 1:** Characteristics of study population at baseline visit classified by sex.

	**Women**	**Men**	**Total**
	***n* = 579**	***n* = 505**	***n* = 1084**
Fungiform papillae density, number/cm^2^	16.14 ± 9.54	13.77 ± 8.61	15.04 ± 9.19
**Demographics**			
Age, years	66.85 ± 13.78	69.04 ± 14.61	67.86 ± 14.20
Follow-up time, years^*^	4.28 ± 1.69	4.19 ± 1.72	4.24 ± 1.70
Number of visits^*^	3.02 ± 1.14	3.07 ± 1.23	3.04 ± 1.18
Whites, %	62.52	72.08	66.97
**Anthropometric**			
BMI^c^, kg/m^2^	26.93 ± 5.17	27.56 ± 4.1	27.23 ± 4.71
% Obese (BMI >30kg/m^2^)	25.04	23.76	24.45
Waist hip ratio	0.80 ± 0.07	0.93 ± 0.07	0.86 ± 0.10
**Smoking**			
Smokers, %			
-Never smokers	68.35	62.82	65.77
-Quit more than 10 years ago	27.48	33	30.06
-Quit less than 10 years ago	3.13	1.79	2.5
-Current smoker	1.04	2.39	1.67
Pack years^a^	4.08 ± 9.84	8.26 ± 17.96	6.03 ± 14.36
**Alcohol**			
Used alcohol in past 12 months, %	79.48	85.17	82.12
Alcoholic drinks in a typical week, continuous^b^	0.96 ± 1.14	1.43 ± 1.35	1.18 ± 1.26
**Alcoholic drinks in a typical week, categorical^b^**			
0 to 1	71.55	54.51	63.62
2 to 3	26.18	39.08	32.18
4 or more	2.27	6.41	4.2

Values reported are mean ± SD or percentages. ^*^Among those with repeat visits. ^a^Among smokers. ^b^Among those who drink alcohol. ^c^BMI: body mass index.

In cross-sectional analysis (Table 2), fungiform papillae density was lower by 1.81 papillae/cm^2^ for every decade of baseline age (*p* < 0.001). Men had a lower fungiform papillae density than women independent of age (β = −1.840, *p* = 0.001). Age and sex adjusted cross-sectional comparisons at baseline showed that Whites had a lower fungiform papillae density (β = −1.435, *p* = 0.012) than non-Whites. A lower fungiform papillae density was associated with a higher waist hip ratio (β = −8.525, *p* = 0.029), a smoking status of being a current smoker (β = −5.133, *p* = 0.014) or having quit within 10 years (β = −3.693, *p* = 0.031) compared to never smokers, and alcohol use within the past 12 months (β = −1.571, *p* = 0.025). BMI expressed either as continuous or categorical, pack years of cigarette use, and number of drinks in a week expressed either as continuous or categorical were not associated with fungiform papillae density at baseline (all *p* > 0.05).

**Table 2:** Cross-sectional associations with fungiform papillae density at baseline.

	**β**	***P* value**
**Demographics**		
Age	–0.181	<0.001
Sex (ref = women)	–1.840	0.001
Race (ref = non-White)	–1.435	0.012
**Anthropometric measures^a^**		
Body mass index^b^	0.076	0.178
Obesity (BMI > 30kg/m^2^)	0.653	0.211
Waist hip ratio	–8.525	0.029
**Smoking measures**		
Smoking status^c^		
Never smokers	Ref	Ref
Quit more than 10 years ago	–0.675	0.258
Quit less than 10 years ago	–3.693	0.031
Current smoker	–5.133	0.014
Pack years of smoking	–0.023	0.220
**Alcohol use measures**		
Used alcohol in past 12 months	–1.571	0.025
Alcoholic drinks in the past 12 months, continuous	-0.093	0.675
**Alcohol drinks in the past 12 months, categorical**		
0 to 1	Ref	Ref
2 to 3	–0.689	0.251
4 or more	0.525	0.699

^a^All models were adjusted for age and sex. ^b^Separate age and sex adjusted models were created for each of the variables listed above. ^c^Smoking status was analyzed as an indicator variable, with never smokers as the baseline category.

**Figure 2:**
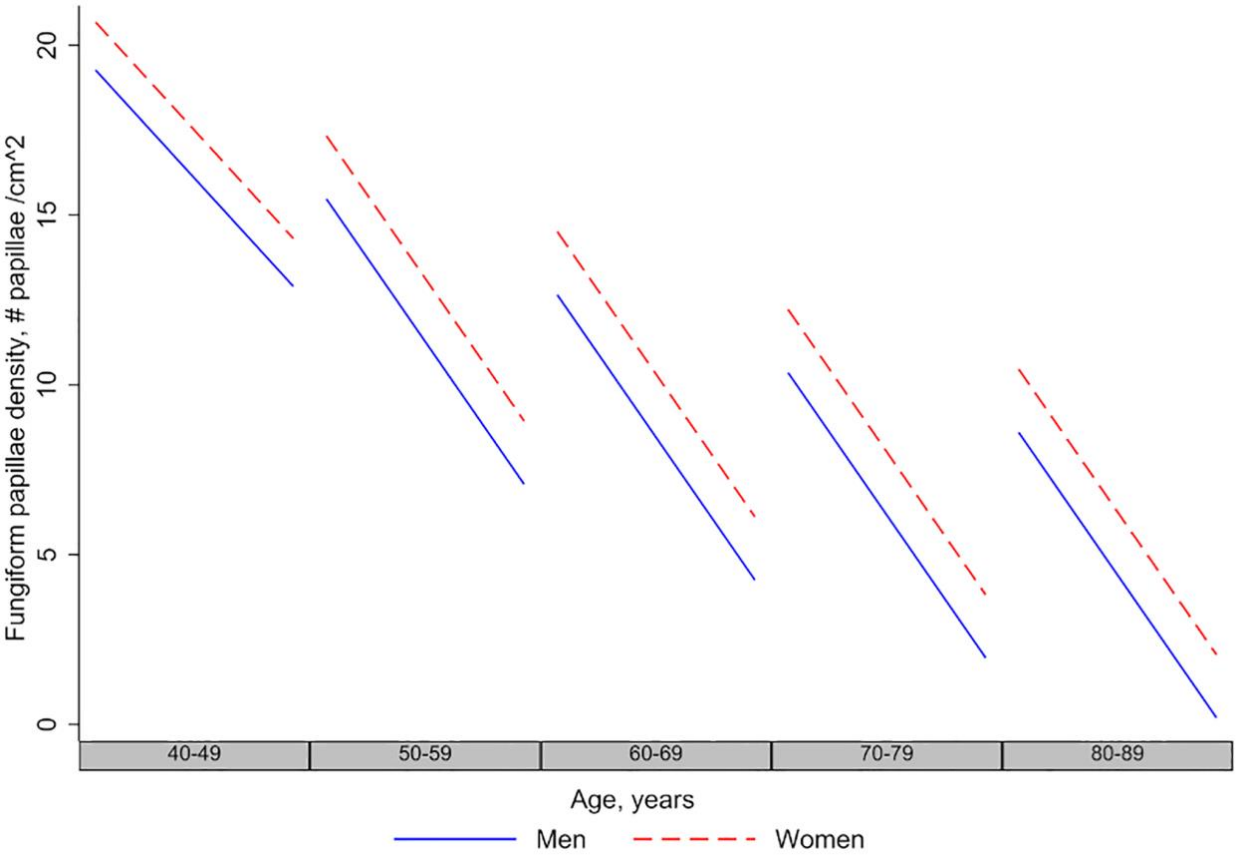
**Longitudinal changes in fungiform papillae density in men and women.** The figure shows that men and women have decreasing fungiform papillae density over age and time, and the longitudinal rate of decline in men and women is the same.

In longitudinal models (Table 3, Model 1), fungiform papillae density decreased with time of follow-up at a rate of approximately 6.46 papillae/cm^2^ per decade (*p* < 0.001). There was no interaction between the baseline age and time variable, indicating that the rate of decline was similar at all ages. The rate of decline in men and women over follow-up time was similar (Figure 2), indicated by the non-significant Sex X Time interaction term (*p* > 0.05). Similarly, interaction of race with time was not significant (*p* > 0.05), indicating that the decline in fungiform papillae density over time did not differ by race.

**Table 3:** Mixed effects models showing longitudinal associations of anthropometric measures.

	**Model 1**		**Model 2a**		**Model 2b**		**Model 2c**	
	**Base model**		**BMI**		**Obesity**		**Waist hip ratio**	
	**β**	***P* value**	**β**	***P* value**	**β**	***P* value**	**β**	***P* value**
Entry age	–0.181	<0.001	–0.166	<0.001	–0.180	<0.001	–0.176	<0.001
Time	–0.646	<0.001	–0.502	<0.001	–0.645	<0.001	–1.596	0.001
Sex (ref = women)	–1.342	0.002	–5.077	0.006	–1.347	0.002	–0.794	0.107
Race (ref = non-White)	–1.869	<0.001	–1.739	<0.001	–1.798	<0.001	–1.857	<0.001
**Anthropometric measures X Time**								
BMI			–0.750	0.453				
Obesity (BMI >30 kg/m^2^)					–0.011	0.933		
Waist hip ratio							1.057	0.057
Constant	29.207	<0.001	27.777	<0.001	28.933	<0.001	34.505	<0.001

Entry age is the age at baseline. BMI: body mass index. ‘X’ indicates an interaction term.

None of the anthropometric measures had a significant interaction with follow-up time (all *p* > 0.05), which means that they did not affect the fungiform papillae density rate of decline. Similarly, none of the smoking variables and none of the alcohol use variables affected the rate of decline, either tested in the model one at a time or together in a multivariate model (Tables 4–6).

**Table 4:** Mixed effects models showing longitudinal associations of smoking measures.

	**Model 1**		**Model 3a**		**Model 3b**	
	**Base model**		**Smoking status**		**Pack years**	
	**β**	***P* value**	**β**	***P* value**	**β**	***P* value**
Entry age	–0.181	<0.001	–0.183	<0.001	–0.179	<0.001
Time	–0.646	<0.001	–0.656	<0.001	–0.670	<0.001
Sex (ref = women)	–1.342	0.002	–1.337	0.002	–1.304	0.003
Race (ref = non-White)	–1.869	<0.001	–1.908	<0.001	–1.866	<0.001
**Smoking measures X Time**						
Smoking status						
Never			Ref	Ref		
Quit > 10 years ago			–0.028	0.822		
Quit < 10 years ago			0.565	0.335		
Current smoker			–0.078	0.893		
Pack years					0.004	0.350
Constant	29.207	<0.001	29.571	<0.001	29.142	<0.001

Entry age is the age at baseline. ‘X’ indicates an interaction term. Since we were interested mainly in the longitudinal effects over time, only the time interaction terms for the smoking measures are reported in the table above. Separate models were created for each of the smoking measures – one model with the categorical smoking status, and one model with the continuous variable Pack years.

**Table 5:** Mixed effects models showing longitudinal associations of alcohol use measures.

	**Model 1**		**Model 4a**		**Model 4b**		**Model 4c**	
	**Base model**		**Alcohol use or not**		**Drinks in a week (continuous)**		**Drinks in a week (categorized)**	
	**β**	***P* value**	**β**	***P* value**	**β**	***P* value**	**β**	***P* value**
Entry age	–0.181	<0.001	–0.182	<0.001	–0.168	<0.001	–0.168	<0.001
Time	–0.646	<0.001	–0.550	<0.001	–0.663	<0.001	–0.654	<0.001
Sex (ref = women)	–1.342	0.002	–1.344	0.002	–1.481	0.033	–1.495	0.002
Race (ref = non-White)	–1.869	<0.001	–1.782	0.006	–1.463	0.002	–1.465	0.005
**Alcohol use measures X Time**								
Alcohol use, yes or no			–0.120	0.414				
Drinks in a typical week, continuous					0.004	0.940		
Drinks in a week, categorical								
0 or 1							Ref	Ref
2 or 3							0.049	0.695
4 or more							–0.072	0.797
Constant	29.207	<0.001	27.933	<0.001	23.151	<0.001	29.13911	<0.001

Entry age is the age at baseline. ‘X’ indicates an interaction term. Since we were interested mainly in the longitudinal effects over time, only the time interaction terms for the alcohol use measures are reported in the table above. Separate models were created for each of the alcohol use measures – one model for “Alcohol use, yes or no”, one model for “Drinks in a typical week, continuous”, and one model for “Drinks in a week, categorical”.

**Table 6:** Mixed effects model showing multivariate longitudinal associations.

	**Multivariate model**	
	**β**	***P* value**
Entry age	–0.183	<0.001
Time	–0.289	0.396
Sex (ref = women)	–1.357	0.002
Race (ref = non-White)	–1.776	<0.001
BMI	0.055	0.305
BMI x Time	–0.010	0.371
**Smoke history**		
Former smoker	Ref	Ref
Quit > 10 years ago	–0.419	0.432
Quit < 10 years ago	–1.925	0.220
Current smoker	–4.099	0.037
**Smoke history X Time**		
Former smoker	Ref	Ref
Quit > 10 years ago	0.004	0.973
Quit < 10 years ago	0.596	0.310
Current smoker	–0.073	0.899
Alcohol use	–0.182	0.759
Alcohol use X Time	–0.123	0.410
Constant	28.121	<0.001

BMI: body mass index.

## DISCUSSION

We provide the first demonstration that fungiform papillae density in humans decreases with age both cross-sectionally and longitudinally in a large, well characterized community-based cohort. Cross-sectionally, fungiform papillae density is lower in men, those with a higher waist hip ratio, and those who smoke and/or drink alcohol. Longitudinally, fungiform papillae density decreases over time. The rate of decline of fungiform papillae density is similar among men and women, and similar among Whites and non-Whites. The longitudinal decline in fungiform papillae density is not affected by obesity, smoking, or alcohol use. Therefore, we conclude that decline in fungiform papillae density is an age-related phenomenon.

### Fungiform papillae density

To our knowledge, the Beaver Dam Offspring Study is the only large cohort study that has investigated the cross-sectional association of age with fungiform papillae density. The Beaver Dam Offspring Study showed a decrease in fungiform papillae density with age in 2371 participants (4). Prior studies on fungiform papillae involved smaller number of subjects (number ranged from 12 to 182) with a narrow age-range (18 to 35 years of age) [33–36]. Since fungiform papillae density varies widely among individuals (Figure 1B), as noted in other studies [4, 33–37], these small studies were unable to reliably estimate the effect of aging on fungiform papillae density in the general population. The mean fungiform papillae density for BLSA cohort is 15.04 ± 9.19 papillae/cm^2^ with a range of 0–67 papillae/cm^2^. Our fungiform papillae counts, obtained using the position we selected for counting the fungiform papillae – approximately 7mm posterior to the apex of the tongue and 7mm lateral to the median sulcus (Figure 1A), do match the counts obtained from autopsy tongue tissues. Specifically, Cheng and colleague sectioned the tongue into 1cm segments; the fungiform papillae density in the first two segments starting from the apex of the tongue, similar in position to where we placed our templates, is approximately 17 papillae/cm^2^ [38]. Miller also showed a decrease in fungiform papillae density from apex to mid-region of the tongue [3]. The wide discrepancy in fungiform papillae density among different studies is most likely due to the location that was used to quantify the fungiform papillae. Studies using area at the apex of the tongue, such as the Beaver Dam Offspring study, yielded higher counts, and studies using tongue region more posterior to the apex yielded lower counts. In addition, studies with a younger cohort would likely yield a higher count as fungiform papillae density has been shown to decrease with age [4]. Our BLSA study cohort, with its wide range of participant age, can provide a more accurate estimate of the aging effect.

### Decline with age

To our knowledge, this is the first report of longitudinal trajectories of fungiform papillae density with age. While other studies have reported cross-sectional changes of fungiform papillae density across different ages, they are not able to provide information on the rate of change over time and are subjected to bias from time-invariant unmeasured confounders and cohort effect. In contrast, longitudinal data can provide us with the precious opportunity to distinguish effects of aging from cohort effects. Reports on the association of fungiform papillae density with age have generally shown a lower fungiform papillae density at older ages [3, 4, 12]. We also report a lower fungiform papillae density at older ages, and additionally demonstrate decline of fungiform papillae density over time.

Many reasons can explain the longitudinal decline of fungiform papillae density. Fungiform papillae are probably subject to the same aging-related pathologies observed in other tissues, such as on-going low-grade inflammation, deficient or defective repair after cellular insults, and decreased and defective replacement of cells from a stem cell population. The fact that fungiform papillae have the potential to be directly exposed to noxious stimuli such as hot, spicy, or pungent food may increase the likelihood of such insults. Fungiform papillae are innervated by the chorda tympani nerves, and have a rich blood supply; declines in the health of these systems with aging could contribute to the aging associated decline in fungiform papillae density [2]. Subjects with taste dysfunction were found to have flat and irregular fungiform papillae with poor blood flow [39]. Transection of the chorda tympani nerve is associated with atrophy of fungiform papillae, which begin to resemble filiform papillae [40], as well as lower fungiform papillae density [6, 11]. Loss of amiloride sensitive neural responses in Skn-1a-deficient mice was correlated with the disappearance of taste receptor cells [41]. Diseases with abnormal neurotrophic support, such as Stüve-Wiedemann syndrome and Machado-Joseph Disease, exhibit taste dysfunction and lower numbers or complete absence of fungiform papillae [42]. By observing the fungiform papillae of all these BLSA participants, the authors noted that the morphology and size are highly variable between individuals, and the distinction between different types of papillae, fungiform versus filiform for example, become less obvious with older age.

Therefore, with fungiform papillae possibly being endocrine organs, decrease in the density or number of fungiform papillae might be an indicator of health for multiple cellular systems at the local level, as well as body systems such as the cardiovascular, peripheral nervous system, endocrine system, or metabolic system; this raises the question of whether fungiform papillae density and taste function could be a marker of aging. Interestingly, certain haplotypes of the TAS2R38 receptor have recently been found to be associated with longevity [43].

### Sex differences

Women had a higher fungiform papillae density than men. We also report for the first time that the rates of decline of fungiform papillae density over time is similar in men and women. Previous reports on the associations between sex, taste, and fungiform papillae density have shown conflicting findings. Some studies have reported no sex related differences in fungiform papillae density [29] or taste [44], while others did find such differences [4, 6, 28]. Women have been shown to have higher taste sensitivity, and supertasters may be more common among women [34]. EGM thresholds have been shown to be lower in young women [6].

The etiology of the sex differences is not clear. It could be related to the sex hormones estrogen and progesterone, as demonstrated by changes in taste intensity and hedonic preferences in pregnancy, such as an increase in bitter intensity [45]. Sex steroid hormones have been shown to affect gustatory processing at the levels of the taste receptor, peripheral nerve, and the central nervous system, with receptors for sex hormones prominently present in several nuclei associated with central gustatory pathways [46].

### Racial differences

Whites had a lower fungiform papillae density than non-Whites, but both groups have a similar rate of decline over time. To our knowledge, racial differences in fungiform papillae density have never been reported previously. However, there have been reports of racial differences in taste sensitivity [47]. Non-Hispanic Blacks have been noted to have a higher prevalence of taste impairment [17], but have higher-rated taste sensations [30]. Taste impairment may be associated with a compensatory increase in fungiform papillae density to help with taste sensation. Proposed mechanisms for these differences include genetic differences such as differences in the frequency distribution and functional variants of TAS2R16 and TAS2R38 haplotypes [30, 48], as well as higher rates of nerve impairment among African Americans due to various factors such as trauma or upper respiratory infections [30]. It is possible that at least some of the effects of these factors on taste perception are mediated via fungiform papillae density, or that these factors could affect fungiform papillae density directly.

### BMI and waist hip ratio

In our study, BMI and obesity (defined as BMI > 30 kg/m^2^) were not associated cross-sectionally or longitudinally with fungiform papillae density. Central obesity measured by waist hip ratio was cross-sectionally associated with lower fungiform papillae density, but had no longitudinal associations with fungiform papillae density. The discrepancy between BMI and waist hip ratio might be due to the fact that waist hip ratio is a better indicator of central or visceral obesity and overall risk than BMI [49]. Additionally, among older adults, a lower BMI might be an indicator of poor nutritional status rather than being a marker of obesity [50]. Previous reports exploring the association between obesity and fungiform papillae density have been conflicted. Proserpio et al. reported a lower fungiform papillae density in those who have a BMI ≥ 30 kg/m^2^, but the study was cross-sectional, with a small sample size and narrow age range [31]. Additionally, no adjustments were made for age differences among the subjects. Fischer et al. did not find an association between obesity and fungiform papillae density in a large population-based cross-sectional study that also used BMI ≥ 30 kg/m^2^ as the measure of obesity [4].

Kaufman et al. found that mice fed with a high fat diet became obese and had a lower fungiform papillae density [51]; this association appeared to be mediated via inflammation, specifically TNF-alpha. However, studies showed mixed results when conducted among humans. A higher BMI was not associated with a lower fungiform papillae density. On the other hand, a study using a cohort of college-aged students found that an increase in neck circumference, taken as surrogate biomarker of obesity, was associated with a longitudinal decrease in fungiform papillae density [8]. However, neck circumference could be affected by other factors such as alcohol use and could be an indicator of other disease conditions such as sleep apnea or cardiovascular disease, which may also affect fungiform papillae density. In addition, the age range of the college-aged cohort studied was narrow. Finally, there are significant differences between the biological systems for taste in mice and men. In our study, we examined a large number of participants from a broad age range, adjusting for age, sex, and race. We also examined obesity in a variety of ways, using indicators of overall obesity as well as central adiposity, and using continuous as well as categorical measures. Our participants also had a broad range of BMI and waist hip ratios.

While obesity may not be related to fungiform papillae density over time, it is still possible that the reverse is true i.e., that fungiform papillae density may be predictive of weight gain over time, and this needs to be examined in future studies. fungiform papillae density has been shown to be associated with taste intensity [3, 10], and it seems plausible that taste and resulting dietary preferences could be associated with weight gain and obesity over time. While choosing a healthy diet high in fruits and vegetables is associated with smaller gains in BMI and waist circumference [52], those prone to obesity may have a heightened hedonic response to unhealthy foods such as sweet and salty food [16, 53, 54], or energy dense foods in general [31].

### Smoking

Current smokers and those who quit less than 10 years ago had a lower fungiform papillae density cross-sectionally in our study. This is in contrast to some previous studies [55], but consistent with more recent studies [4, 29]. Smokers have also been shown to have higher electrogustatory thresholds and lower taste sensitivity than non-smokers [16].

However, none of the various smoking measures that we used were associated with longitudinal changes in fungiform papillae density. Nevertheless, these results should be considered with caution because the BLSA population tend to be healthy, with a low prevalence of smokers and especially, of heavy smokers. Therefore, the lack of significant longitudinal association could be due to the small proportion of smokers that we had, although the association we observed cross-sectionally with categories of smoking status would argue against that. It is also possible that the reverse is true in that fungiform papillae density, through its associations with taste intensity, could be associated with changes in smoking preferences over time.

### Alcohol use

Alcohol consumption within the past 12 months was associated with lower fungiform papillae density cross-sectionally in our study. The number of drinks per day was not associated with fungiform papillae density cross-sectionally. Additionally, none of the alcohol use variables were associated with longitudinal changes in fungiform papillae density. Again, this could be due to the small proportion of heavy drinkers in our study. In the study by Fischer et al., fungiform papillae density was lower among those consuming 4 or more drinks per day [4], but in our study, those consuming 4 or more drinks were less than 2 percent of the study population.

### Strengths and limitations

The strengths of our study include a well characterized cohort of over 1000 participants with wide age-range and multiple follow-up visits. More importantly, this is the first study to follow fungiform papillae density longitudinally and the first to provide a precious opportunity to distinguish effects of aging from cohort effects. However, our study has limitations. The morphology of the fungiform papillae varies among individuals and distinction between fungiform papillae and other papillae becomes less obvious with age, which may introduce variability into the counting of fungiform papillae.

## CONCLUSIONS

Fungiform papillae density decreases longitudinally over time. Further studies are needed to explore other possible determinants of the longitudinal decline in fungiform papillae density. It is also important to know whether the observed decline in fungiform papillae density leads to effects such as modifications in dietary patterns, smoking or alcohol use, and morbidity. As potential endocrine organs, fungiform papillae may provide us with a new window into human physiology that is easily accessible and observable, one that could be utilized in biopsy for further testing and examination. Along those lines, we have recently reported that SARS-CoV-2 infect taste receptor cells in taste buds of biopsied fungiform papillae [56]. With all these relevant areas of research in mind, we have developed an *in vivo* model and mechanistic studies are currently underway to study age-related and SARS-CoV-2 related taste loss or changes. In summary, fungiform papillae density may potentially serve as an indicator of aging and an indicator for endocrine- and disease-related processes.

## METHODS

### Study population

Participants were selected from the BLSA, a study of normative human aging established in 1958, with a well-characterized cohort, and currently conducted by the Intramural Research Program of NIA. Participants are followed for life every 1 to 4 years, depending on age. The BLSA protocol was approved by the Institutional Review Board of the National Institutes of Health and written informed consent was obtained from all participants at each visit. Eligible participants for this study had to have measures of fungiform papillae density, and have anthropometric measures detailed below.

### Fungiform papillae density

In the BLSA, we began to quantify fungiform papillae density in 2011 using modified procedure originally developed by Miller and Reedy [33]. Briefly, blue food coloring is used to provide optimal contrast between fungiform papillae (which do not take up blue dye and appear pink) and other tongue structures (coated blue). Two clear plastic hole reinforcement templates (7 mm inner diameter) are placed posterior to the apex of the tongue with one template on each side of the median sulcus (Figure 1A). Tongue images containing the two templates were taken using a digital camera. The fungiform papillae present within the two 7 mm holes were then counted and normalized to the area of the holes and expressed as fungiform papillae density (number of fungiform papillae/cm^2^).

### BMI and waist hip ratio

Weight was measured in kilograms and height in centimeters. Body mass index (BMI) was calculated as weight in kilograms divided by the square of height in meters. BMI was used both as a continuous variable, as well as a categorical variable, defined as either obese (BMI > 30 kg/m^2^) or not obese (BMI ≤ 30 kg/m^2^). Waist circumference was measured as the minimal abdominal circumference between the lower edge of the rib cage and the iliac crests. The hip was defined as the maximal circumference around the gluteal muscles below the iliac crests. Waist hip ratio was then calculated, as a continuous measure, by dividing waist circumference by hip circumference.

### Smoking status

Smoking status was examined two different ways. One method is to defined smoking using a categorical variable – “never smoker”, “quit more than 10 years ago”, “quit less than 10 years ago”, or “current smoker”. We also coded smoking as pack years of smoking, calculated as average number of packs of cigarettes smoked multiplied by years of use. Those who had never smoked were coded as 0 pack years.

### Alcohol use measures

At each visit, participants were asked if they had used alcohol within the past 12 months. This was used as a binary variable consisting of “Yes” or “No” responses. If they had used alcohol, they were asked how many drinks they had on average each day, and this was then categorized in to 3 categories – “0 or 1”, “2 or 3”, and “4 or more”. Those who had not used alcohol within the past 12 months were coded as 0 drinks per day.

### Statistical analysis

First, we compared baseline characteristics between men and women using Student’s *t*-tests and chi-squared tests for continuous variables and categorical variables, respectively. Then we examined the baseline cross-sectional associations of fungiform papillae density with various covariates using age- and sex-adjusted linear regression models. Finally, we tested for longitudinal changes and determinants of fungiform papillae density using linear mixed effects models, which can handle unbalanced, unequally spaced observations that are typical of a longitudinal study such as the BLSA.

Initial longitudinal models tested for the longitudinal changes of fungiform papillae density with baseline age, follow-up time, as well as with sex and race and their interaction terms with follow-up time. Longitudinal trajectories for fungiform papillae density for men and women were graphed using the base model. Subsequently, we tested, one at a time, for any longitudinal associations of the different measures with fungiform papillae density by testing for the significance of their interaction term with time. Finally, we tested the different covariates together in a multivariate model. All analysis was done by Stata Statistical Software (Release 16. College Station, TX: StataCorp LLC. StataCorp. 2017).
